# Shaping the Cortical Landscape: Functions and Mechanisms of Top-Down Cortical Feedback Pathways

**DOI:** 10.3389/fnsys.2020.00033

**Published:** 2020-06-10

**Authors:** Edward Zagha

**Affiliations:** Neuroscience Graduate Program, Department of Psychology, University of California, Riverside, Riverside, CA, United States

**Keywords:** feedback, motor preparation, attention, predictive coding, cortical circuits, functional connectivity

## Abstract

Cortical feedback pathways are proposed to guide cognition and behavior according to context and goal-direction. At the cellular level, cortical feedback pathways target multiple excitatory and inhibitory populations. However, we currently lack frameworks that link how the cellular mechanisms of cortical feedback pathways underlie their cognitive/behavioral functions. To establish this link, we expand on the framework of signal routing, the ability of cortical feedback pathways to proactively modulate how feedforward signals are propagated throughout the cortex. We propose that cortical feedback modulates routing through multiple mechanisms: preparing intended motor representations, setting the trigger conditions for evoking cortical outputs, altering coupling strengths between cortical regions, and suppressing expected sensory representations. In developing this framework, we first define the anatomy of cortical feedback pathways and identify recent advances in studying their functions at high specificity and resolution. Second, we review the diverse functions of cortical feedback pathways throughout the cortical hierarchy and evaluate these functions from the framework of signal routing. Third, we review the conserved cellular targets and circuit impacts of cortical feedback. Fourth, we introduce the concept of the “cortical landscape,” a graphical depiction of the routes through cortex that are favored at a specific moment in time. We propose that the cortical landscape, analogous to energy landscapes in physics and chemistry, can capture important features of signal routing including coupling strength, trigger conditions, and preparatory states. By resolving the cortical landscape, we may be able to quantify how the cellular processes of cortical feedback ultimately shape cognition and behavior.

## Introduction

Bidirectional (feedforward and feedback) signaling is a fundamental organizing principle of the neocortex. The cortical feedforward system originates in primary sensory cortices and connects to higher-order cortical areas. Most studies of the cortex have focused on the transformation of sensory signals along feedforward pathways. And yet, cortical regions connected by feedforward pathways are also connected by an equal or greater density of feedback pathways, connecting higher order to lower-order regions. This dominant, pervasive and fine-grained anatomical feedback is a defining feature of neocortical circuits (Felleman and Van Essen, 1991; Markov et al., 2014). From the principle “form follows function” we believe that understanding the interplay of feedforward and feedback signaling is key to understanding neocortical function.

**Figure 1:**
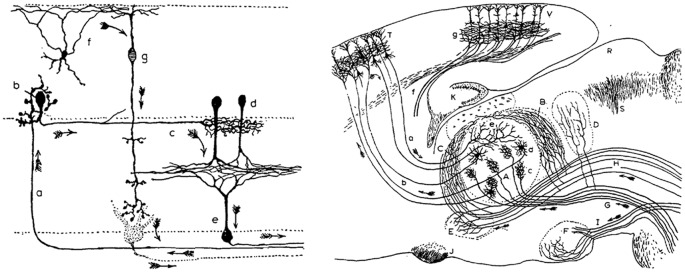
Early descriptions of feedback pathways in subcortical and cortical structures. These drawings of neural circuits by Santiago Ramón y Cajal are among the first depictions of feedback pathways. (Left) Ramón y Cajal’s drawing of retinal circuitry. Arrows in the downward direction (arrows “g” and “e”) reflect feedforward or *centripetal* pathways from the photoreceptors towards higher-order brain structures for the transmission of visual stimuli. Ramón y Cajal additionally noted the presence of feedback or *centrifugal* fibers (arrow “a”) which originate in higher-order brain areas and target the retina. Thus, feedback occurs even at the earliest levels of sensory processing. (Right) Ramón y Cajal’s drawing of pathways of the somatosensory system. Demarcated are feedforward fibers from the brainstem to thalamus (arrow “G”) and from the thalamus to the cortex (arrow “b”). Also illustrated here are feedback fibers from cortex to thalamus (arrow “a”). While feedback fibers appear to be relatively sparse near the periphery, they are highly abundant in the thalamocortical system. In describing this anatomical organization during his Nobel prize speech in 1906, Ramón y Cajal speculated as to the function of feedback: “What is the role of the centrifugal fibres? Are they, as Duval thinks, conductors destined to produce in the sensory pathway articulations a very intimate contact which would be indispensable for the satisfactory propagation of the nervous impulse? Or rather do they transport some form of energy from the brain, the rapid accumulation of which in the sensory stations is necessary for the passage of ascending nerve currents? Unfortunately, at this stage of science, it is impossible to give satisfactory and categorical answers to these questions”. Now, over 100 years later, scientists continue to debate the functions of feedback fibers (panels and quote are reproduced with permission from © The Nobel Foundation: https://www.nobelprize.org/prizes/medicine/1906/cajal/lecture/).

An important starting point of this discussion is to define “feedback,” and disentangle its anatomical vs. functional meanings. Our use of the term “feedback” is exclusively as an anatomical description (Figure 1), within the framework of cortex as a hierarchically organized structure (Felleman and Van Essen, 1991; described in further detail below). The alternative interpretation of “feedback” is the functional sense of responding to feedforward drives and “closing the loop.” For some processes, feedback pathways do contribute to a functional feedback signal. However, for other processes, the relationship is unclear or even reversed. For example, during predictive coding, the functional feedback signal is carried by feedforward pathways (described in further detail below). To avoid confusion, we use the terms “feedback” and “feedback pathways” synonymously, and according to their anatomical definitions.

There are two general lines of research studying cortical feedback. One is aimed at understanding the contributions of cortical feedback to behavior and cognition. These studies are typically performed in awake subjects performing goal-directed tasks or in computational models simulating these experimental conditions. The second line of research is aimed at understanding the cellular targets and circuit impacts of cortical feedback activity. These studies are typically performed using *in vitro* physiology or histology. Despite this two-front effort, our most significant current limitation is in translating between the two: understanding how the cellular and circuit mechanisms of cortical feedback underlie their cognitive and behavioral functions.

The purpose of this manuscript is meant to bridge this gap. Our framework emphasizes the roles of cortical feedback in routing feedforward signals as needed for a specific context. This framework of cortical feedback as modulating feedforward signal routing is not novel in the literature. It is prominent in descriptions of the prefrontal cortex (PFC; Miller and Cohen, 2001) and models of attention (Fries, 2015). Nonetheless, we believe that this manuscript is important for three main reasons. First, we provide a broad overview of feedback functions throughout the cortical hierarchy, and therefore expand the signal routing framework throughout cortex. Second, we provide a broad overview of cortical feedback cellular targets and circuit impacts, which establishes testable hypotheses for how routing may occur. Third, we identify specific directions for future research that emerge from this signal routing framework.

## Importance of Signal Routing in the Neocortex

Consider a simple act of sensory selection. I walk through the produce section of a grocery store to buy carrots. After passing radishes and eggplants, I spot the carrots on a shelf and reach out my arm to grab a few. What neural mechanisms enabled this behavior? The “carrot” sensory signals propagate first to the primary visual cortex, far back in my occipital lobe. To trigger a reaching movement, these visual signals must propagate to the left arm representation of the motor cortex in my frontal lobe, which sits multiple synapses away. How was the carrot stimulus routed to activate my left arm motor cortex (useful), rather than being ignored or routed to activate my right leg motor cortex (not useful)? And how were the radish and eggplant stimuli ignored (useful), rather than also activating my arm motor cortex (not useful)?

The proper routing of cortical signals is essential for goal-directed behavior. As illustrated above, routing is critical for ensuring that the correct trigger stimulus (carrot) initiates the correct motor plan (arm reach). However, routing also determines which sensory streams are attended and which are ignored. Routing determines which percepts to place into working memory and under which conditions. Most generally, routing enables us to perform the task at hand, rather than being overcome by the continuous distractions in our environment.

We propose that cortical feedback pathways modulate feedforward signal routing throughout cortex according to one’s goal-direction (i.e., searching for carrots vs. listening to music), behavioral context (i.e., walking vs. sitting) and environmental context (i.e., at the grocery store vs. at a concert). We emphasize that this function is not “feedback” in the functional sense of responding to feedforward drives. In contrast, we propose that feedback pathways establish the initial conditions for proper routing well before a stimulus arrives. As we will see below, this includes preparing for the deployment of intended actions, setting trigger conditions for evoking cortical outputs, modulating coupling strengths between cortical regions, and suppressing expected sensory representations. Thus, we propose a framework for cortical feedback pathways as *proactive regulators of feedforward signal propagation*. We consider signal propagation within the cortex as a landscape, analogous to energy landscapes commonly found in physics, chemistry, and biochemistry. Preferred stimuli flow down valleys to their intended targets, while non-preferred stimuli are stopped abruptly by high peaks. The cortical landscape changes constantly depending on how our immediate goals, behavior, and surroundings alter the meaning and valence of different stimuli and different actions. We propose that is it the unique and primary function of cortical feedback pathways to shape the cortical landscape, to establish the routes between sensory, cognitive, and motor representations according to our immediate goals.

While signal propagation is common to all neural structures, regulated signal routing is particularly important for neocortex. The neocortex is essential for suppressing impulsive responses and forming new, context-dependent sensory-motor associations (Aron et al., 2003; Chambers et al., 2006; Arnsten and Rubia, 2012; Moore et al., 2012). The ability to form rapid, arbitrary sensory-motor associations has two important implications. (1) Dense connectivity: if any sensory signal may be paired with any motor response, there must exist near-complete (albeit, indirect) connectivity between all sensory and all motor representations. (2) Dynamic connectivity: given dense connectivity throughout the cortex, these anatomical pathways must be heavily regulated to ensure the pairing of useful associations and the suppression of all other associations. Moreover, this dynamic regulation must occur on the timescale of 100s of milliseconds, to track our ongoing changes in goal-direction (what is the next item on my shopping list?). Dense connectivity allows for the visual representation of “carrot” to trigger a wide diversity of potential responses. Dynamic connectivity ensures that the selected response is the one appropriate for achieving my immediate goals.

### What Are Cortical Feedback Pathways?

**Figure 2:**
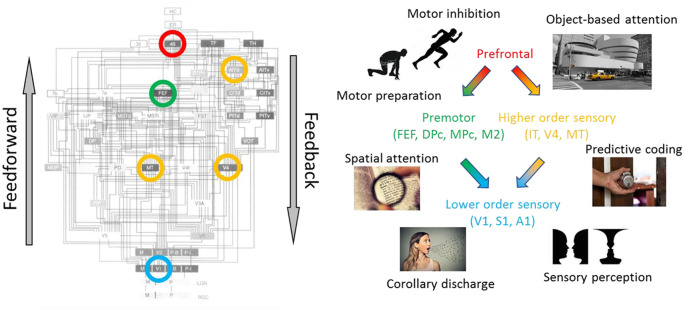
Diverse functions of cortical feedback throughout the hierarchy. (Left) Hierarchical organization of the primate visual system adapted with permission from Felleman and Van Essen (1991). Circled regions are some of the cortical regions discussed in the manuscript. (Right) Proposed functions of cortical feedback, segregated according to source and the target region. (Red) prefrontal cortex (PFC): Brodmann area 46, dorsolateral PFC; (green) premotor cortices: FEF, frontal eye field; DPc, dorsal premotor cortex; MPc, medial premotor cortex; M2, secondary motor cortex; (yellow) higher-order sensory cortices: IT, infratemporal cortex; V4, visual area V4; MT, middle temporal visual area; (blue) lower-order sensory cortices: V1, primary visual cortex; S1, primary somatosensory cortex; A1, primary auditory cortex.

Felleman and Van Essen (1991) presented an iconic mapping of the primate visual system (Figure 2). Their anatomy-based mapping emphasized a hierarchical organization, such that two brain regions could either be at the same organizational level or one could be “lower-order” and the other “higher-order.” This ordering begins (lowest) near the sensory receptors. The ordering continues to primary sensory, higher-order sensory, motor, and prefrontal cortical regions, and ends (highest) with the hippocampal system. Within this hierarchical organization “feedforward pathways” refers to connections from a lower-order to a higher-order region (e.g., primary sensory cortex to secondary sensory cortex). Most generally, feedforward pathways convert raw sensory inputs into unified sensory percepts and motor commands. For example, the (V1→V2→V4→IT) pathway transforms simple spatial filtering into discrete object representations for object recognition, whereas the (V1→V2→MT→FEF) pathway transforms the same input into motion representations to guide eye movements (Maunsell and Newsome, 1987).

In contrast to feedforward pathways, “feedback pathways” refers to connections from higher-order to lower-order regions (Rockland and Pandya, 1979). Feedback pathways are more numerous than feedforward pathways (Markov et al., 2014), suggesting that they make essential contributions to cortical function. Cortical feedback originates from pyramidal neurons, which project glutamatergic feedback axons that depolarize their post-synaptic targets (Felleman and Van Essen, 1991; Johnson and Burkhalter, 1994; Shao and Burkhalter, 1996; Rocco and Brumberg, 2007; Petreanu et al., 2009). For many pathways the cellular targets of cortical feedback are unknown. Where they have been studied, feedback axons are found to synapse onto pyramidal neurons and GABAergic interneurons (Gonchar and Burkhalter, 2003; Anderson et al., 2011; Lee et al., 2013; Zagha et al., 2013; Kinnischtzke et al., 2014).

Two cortical regions separated by a single hierarchy are often reciprocally connected with feedforward and feedback connections (Felleman and Van Essen, 1991; Markov et al., 2014). However, there are notable anatomical differences between these pathways. Feedforward pathways predominantly originate from layer 2/3 pyramidal neurons and project preferentially to middle cortical layers (layers 4 and 3) in their target region. Feedback pathways predominantly originate from layer 5a pyramidal neurons and project preferentially to superficial (layer 1) and deep (layers 5 and 6) layers. For a detailed characterization of cortical feedforward and feedback anatomy well beyond this general description, see Markov et al. (2014). Importantly, this anatomically-defined hierarchy also reflects functional differences between cortical regions. As one moves up the cortical hierarchy, sensory responses occur at longer latency, have larger receptive field size, persist for a longer duration, and represent increasing stimulus complexity (Maunsell and Newsome, 1987; Murray et al., 2014; Siegle et al., 2019).

Any input to cortex may influence feedforward signal routing. Moreover, ascending neuromodulatory pathways (such as those releasing norepinephrine, dopamine, serotonin or acetylcholine) have traditionally been proposed as the primary regulators of feedforward signal propagation (Lee and Dan, 2012; Zagha and McCormick, 2014). What makes cortical feedback unique? Complex, goal-directed behavior requires exquisitely fine-tuned and rapid regulation of sensory and motor processing. Cortical feedback is the only neural system with the representational complexity, target resolution, and speed capable of implementing such regulation. Regarding representational complexity, a neural system can only signal at the resolution of its informational content. For example, a system that drives attentional shifts to a specific region of space (30 degrees to the left of fixation) or a specific object (carrots) must itself possess representation at equivalent spatial or object resolution. Since the cortical feedforward and feedback systems overlap, they have equivalent representational complexity [particularly if we also consider feedback from primary sensory cortices to thalamic relay nuclei (Singer, 1977; Sherman and Guillery, 1996)]. With matching informational content, cortical feedback is uniquely suited to regulate feedforward signal propagation. As for target resolution, feedback pathways not only connect two cortical regions but do so with receptive field specificity. Feedback pathways travel at layer 6—white matter border, then ascend into the gray matter in which they innervate (Veinante and Deschênes, 2003). Feedback axons ramify into discrete patches of synaptic terminals in deep cortical layers (Salin et al., 1995; Angelucci et al., 2002) before reaching layer 1 where they extend laterally and innervate broadly. In the mouse visual system, retinotopic alignment was recently tested at the level of individual axon terminals in layer 1 (Marques et al., 2018). Receptive fields of cortical feedback axon terminals on average aligned with their target region. Interestingly, conditions of receptive field mismatch were not random but correlated with the tuning properties of the feedback terminals. These data demonstrate the possibility of highly structured spatial- and feature-specific signaling along feedback pathways. Receptive field alignment has also been demonstrated functionally in the primate visuomotor system, between the movement field of FEF neurons and the visual receptive field of V4 neurons (Moore and Armstrong, 2003). In addition to high receptive field resolution, cortical feedback pathways have high temporal resolution [onset time ~10 ms, offset time ~20 ms (Zagha et al., 2013)] consistent with post-synaptic effects mediated by ionotropic glutamate receptors. The representational complexity, target resolution, and speed of the cortical feedback system far exceed what has been demonstrated for ascending neuromodulatory pathways (Metherate et al., 1992; Steriade et al., 1993; Jones, 2003; Goard and Dan, 2009; Sarter et al., 2009; Pinto et al., 2013).

### How to Study Cortical Feedback Pathways?

Our understanding of cortical feedback processing is much less developed than our understanding of feedforward processing. We identify specific challenges in studying cortical feedback. First, cortical feedback lacks a well-defined “stimulus set.” Feedforward processing is initiated with sensory stimulation, for which there are ample stimulus sets with clear parameters (even if the precise details of these parameters are constantly under debate). Does cortical feedback have an analogous stimulus set? We argue below that cortical feedback processing begins with goal-direction. Accordingly, we consider *behavioral task design* to be the relevant stimulus set for cortical feedback. Of course, this means that studying endogenous cortical feedback processing must occur in subjects performing goal-directed tasks with tractable internal states. This requirement complicates the application of many experimental tools that are designed for reduced (e.g., anesthetized or *in vitro*) preparations.

A second challenge is the difficulty in studying cortical feedback with high specificity. A common approach to studying cortical feedback is to stimulate or suppress a higher-order cortical region and measure impacts on behavior or activity in a lower-order cortical region. While this approach has been highly informative it is not specific, as each cortical region sends out multiple feedforward, feedback, and lateral projections. Thus, any observed behavioral or physiological effects from cortical stimulation or suppression cannot be uniquely ascribed to any single projection pathway. Another approach has been to use certain electrophysiological features to infer cortical feedback activity. A negative-going evoked potential measurable by electroencephalogram (EEG) termed the N100 (or N1) has temporal and spatial (laminar) characteristics consistent with mediation by cortical feedback (Cauller and Kulics, 1991). Other studies report that feedforward and feedback signaling have distinct spectral characteristics, with intra-cortical synchronization occurring in the gamma-band (60–80 Hz) along feedforward pathways and the beta-band (14–18 Hz) along feedback pathways (Bastos et al., 2015). However, neither method is fully separable, making it impossible to reliably isolate cortical feedback from feedforward, lateral, and local electrophysiological sources. One electrophysiological approach does achieve high specificity. By pairing single-unit electrophysiological recordings with antidromic stimulation (collision test), researchers have been able to identify cortical feedback projecting neurons and track their activity during the behavioral performance (Beloozerova et al., 2003a,b; Li et al., 2015; Merrikhi et al., 2017). However, this approach is low throughput and technically very challenging.

New tools are making it possible to study cortical feedback at high specificity and resolution. In general, these strategies leverage knowing both the source and target of a specific pathway. One may use an anterograde virus to express a sensor or effector transgene within a higher-order cortex. This will drive expression in all projection pathways. However, specificity may be achieved by imaging (sensor) or stimulating (effector) proteins only in cortical feedback axons and terminals within the lower-order region (Petreanu et al., 2012; Kwon et al., 2016). Petreanu et al. (2012) used this approach to record GCaMP Ca^2+^ activity in the motor to sensory cortical feedback axons and terminals in mice performing a whisker localization task. An alternative strategy is to use a retrograde virus to express a sensor or effector transgene within the lower-order cortex and image/stimulate in somatodendritic compartments within the higher-order region. Causal experiments require additional considerations. Stimulation, even if restricted to cortical feedback axons, will propagate and cause “off-target” activations. Pathway specificity can only be achieved by suppressing cortical feedback axons and terminals (Mahn et al., 2016), as suppression does not actively propagate. Studies in mouse (Manita et al., 2015) and non-human primates (Nurminen et al., 2018) have used optogenetic suppression of cortical feedback axons and terminals to achieve pathway specificity. By implementing these new approaches in subjects performing well-controlled goal-directed tasks, we are primed to make many discoveries about cortical feedback signaling. The recording methods will reveal the representational correlates and potential mechanisms of feedback pathways whereas optogenetic suppression will reveal the necessity of these pathways for specific behaviors.

## Propagating Contexts Through Cortical Feedback

The functions of cortical feedback depend on the representational content of the source and target regions (Figure 2). In the following sections, we group cortical feedback descriptions according to source, target, and function. Within each section, we first introduce specific cognitive or behavioral functions. Second, we highlight the evidence for the involvement of cortical feedback in these functions. Third, we relate these functions to the framework of signal routing. These broad categories are not meant to be a definitive catalog, as future studies will undoubtedly reveal new functions and mechanisms of cortical feedback.

### Prefrontal Cortices to Motor Cortices (Motor Preparation and Impulse Control)

We begin with cortical feedback from PFC. PFC refers to a group of cortical regions rostral to motor cortices with the general function of “executive control”—the processes by which behavior is regulated by goal-direction rather than habit or impulse. Abnormalities in the PFC impair the ability to withhold impulsive responses and form flexible sensory-motor associations (Aron et al., 2003; Chambers et al., 2006; Arnsten and Rubia, 2012; Moore et al., 2012; Hardung et al., 2017). Neurons in PFC are notable for their ability to maintain a persistent representation of the current goal (Fuster and Alexander, 1971; Funahashi et al., 1989; Miller et al., 1996; Compte et al., 2000). However, to exert executive control, the representation of goal-direction within PFC must influence other brain regions. Accordingly, during working memory tasks, content-specific changes in neural activity are not limited to PFC but are observed throughout the cortex (Rose et al., 2016). Cortical feedback pathways are a major route by which PFC regulates sensory and motor processing according to goal direction (for an excellent review of PFC function in top-down executive control, see Miller and Cohen, 2001).

Motor cortices are the cortical regions most directly related to motor control: they initiate movements with low threshold stimulation and express robust motor command signals which predict the content and timing of upcoming movements (Penfield and Rasmussen, 1950; Georgopoulos et al., 1982; Hanes and Schall, 1996). Goal-directed behaviors require the execution of the correct movements at precisely the correct moments. During goal-directed behavior, an intended movement may be planned well ahead of execution (e.g., preparing to run from the starting line of a race once I hear the gunshot). How are goal-directed movements planned? To study motor planning, researchers separate motor preparation from motor execution by including a delay between task instruction and a “go” trigger signal. Task instruction initiates robust changes in spiking activity in motor cortices. This preparatory activity does not initiate movement during the delay but instead accelerates the execution of the instructed movement at the “go” signal (Tanji and Evarts, 1976; Riehle and Requin, 1993; Churchland and Shenoy, 2007; Erlich et al., 2011). Preparatory activity in motor cortices is now understood as establishing the initial conditions for activating a specific motor sequence upon triggering (Vaadia et al., 1988; Churchland et al., 2010).

What is the source of preparatory signals in motor cortices? Evidence points to PFC as sending motor planning signals to motor cortices along feedback pathways. PFC projects to the premotor cortex, which in turn projects to the primary motor cortex (Barbas and Pandya, 1987). To reveal the relationships between these regions, experimenters have recorded neural activity in both prefrontal and premotor cortices during the same task (Boussaoud and Wise, 1993; Muhammad et al., 2006; Cromer et al., 2011). PFC was found to represent multiple aspects of task engagement and performance, whereas the premotor cortex preferentially responded to cues relevant for motor planning (Wang et al., 2019). This is consistent with a hierarchical framework in which the PFC processes information according to task rules and propagates outcomes related to action selection through cortical feedback to premotor cortices to update the motor plan.

Additional processes are needed to prevent motor initiation before it is most optimal (e.g., preventing false starts). Accordingly, a major function of the motor system is to prevent premature motor execution through processes referred to as “behavioral inhibition” or “impulse control.” Studies suggest that PFC to premotor cortical feedback, in addition to signaling motor preparation, also contributes to impulse control. In a delayed-response (waiting) task, Narayanan and Laubach (2006) inactivated PFC while recording from motor cortex neurons. PFC suppression impaired the ability to wait through the delay and increased premature responses. Interestingly, PFC suppression also reduced motor cortex activity during the delay (Narayanan and Laubach, 2006). Computational modeling simulations predict that top-down signals to motor cortices suppress impulsive responses by indirectly inhibiting movement-related neurons (Lo et al., 2009). Thus, PFC to premotor cortex feedback may both set the initial conditions for an upcoming movement and prevent impulsive movement generation. However, direct tests for the involvement of feedback pathways in these processes are currently lacking.

From the perspective of routing, we can describe the contributions of prefrontal to motor cortex feedback as setting the output stage of feedforward signal routing. This includes determining which motor plan to prepare and the trigger conditions (or “energy barrier”) for initiating that plan. Once a feedforward stimulus exceeds the trigger conditions, motor cortices initiate the prepared motor plan. Below we will see how feedback pathways to non-motor areas modulate sensory responses to increase or decrease the probability of exceeding these trigger conditions. In this section, we describe research findings related to motor initiation. However, we speculate that similar processes may relate to other potential outputs of feedforward signaling, such as triggering short-term and long-term memory processes or updating decision-related trigger conditions.

### Prefrontal Cortices to Higher-Order Sensory Cortices (Feature-Based Attention)

Attentional processes bias the feedforward propagation of sensory stimuli according to stimulus properties and goal-direction. Feedback pathways are critical to top-down attention, for which prior knowledge (including goal direction) determines the attentional focus. Importantly, top-down attention can be deployed well before the arrival of a stimulus. As sensory stimuli stream by, attentional processes enhance attended sensory streams and/or suppress unattended sensory streams (Treisman, 1964). Neural correlates of attention have been studied extensively at cellular and population resolution, and many excellent articles have reviewed this topic (Desimone and Duncan, 1995; Kastner and Ungerleider, 2000; Noudoost et al., 2010). In general, the attentional bias of sensory representations increases as one moves up the cortical hierarchy (Moran and Desimone, 1985; Tootell et al., 1998; Buffalo et al., 2010; Aruljothi et al., 2020). For example, internal representations of attended and unattended stimuli may be similar at the level of a primary sensory cortex and yet completely biased in favor of attended stimuli within higher-order sensory cortices.

How does attention cause the selective filtering of attended vs. unattended stimuli? One proposed mechanism is that attention alters the coupling strengths between cortical regions. Coupling strength is the ability of activity in one cortical region to drive activity in a connected cortical region; coupling strength can be inferred by measuring the activity from multiple regions simultaneously and computing their activity correlations (functional connectivity) or modeling their interactions (effective connectivity; Friston, 2011). fMRI and electrophysiological studies have demonstrated changes in coupling strength within cortex with changes in attention (e.g., Buchel and Friston, 1997; Engel et al., 2001; Sakai and Passingham, 2003, 2006; Gregoriou et al., 2009; Al-Aidroos et al., 2012; Briggs et al., 2013; Bastos et al., 2015; Ruff and Cohen, 2016). Generally, coupling strength is enhanced between cortical regions within the attended stream and reduced within unattended streams. Attention-related changes in coupling have even been demonstrated at the synaptic efficacy of single units and the correlation structure of unit pairs (Briggs et al., 2013). Although the details and cellular mechanisms are far from resolved, dynamic regulation of the coupling between cortical regions appears to be a fundamental mechanism underlying top-down attention.

This section highlights feature-based attention which prioritizes one stimulus feature (attended) at the expense of other features (unattended). “Features” can refer to object categories (faces, cars) or sensory components (color, motion). What is the role of PFC feedback in feature-based attention? Through modeling cortical interactions (Buchel and Friston, 1997) and stimulation experiments (Morishima et al., 2009), PFC appears to regulate which sensory pathways are strengthened or weakened. Morishima et al. (2009) demonstrated that stimulating the same prefrontal region differentially impacts posterior visual areas depending on the attentional focus (motion vs. faces). These findings are consistent with a framework in which feature-based attentional signals originate in PFC and propagate through feedback pathways to modulate the coupling strength of task-specific sensory streams. In turn, changes in coupling determine whether a stimulus is attended (high coupling, propagated to higher-order sensory cortices) or ignored (low coupling, prevented from propagating to higher-order sensory cortices).

Within the framework of signal routing, we can appreciate the interplay of feature-based attention and motor preparation/inhibition. As in the grocery store example, feedback-mediated attentional processes ensure that only the internal representation of “carrot” is propagated to the motor cortex with sufficient energy to trigger the prepared action. Through progressive filtering *via* unfavorable coupling, the internal representations of undesired stimuli (radish and eggplant) are attenuated to below the triggering thresholds and therefore are ignored.

### Motor Cortices to Sensory Cortices (Spatial Attention and Corollary Discharge)

In the following sections, we move away from PFC as the source of cortical feedback. Here, we focus on feedback projections from motor to sensory cortices. As described above, motor cortices produce robust peri-movement and preparatory activity. Accordingly, these regions are well-suited to signal ongoing and future motor sequences. The first process we consider is top-down spatial attention, prioritizing one location (attended) at the expense of other locations (unattended). One example from the visual system is attending to a projection screen during a lecture. Spatial attention can enhance the processing of visual stimuli on the screen while reducing the processing of surrounding stimuli. One may *overtly* attend to the screen by physically orienting towards it such that the screen projects directly to the fovea. Alternatively, one may *covertly* attend to a stimulus by orienting one’s attention (i.e., looking at the speaker but attending the screen). Previously it was largely believed that spatial attention and movement are regulated by two separate neural systems. The premotor theory of spatial attention (Rizzolatti et al., 1987) proposed that the two are intimately connected. Overt attention is mediated by a supra-threshold activation of the motor system; covert attention is mediated by a sub-threshold activation of the same motor system.

**Figure 3:**
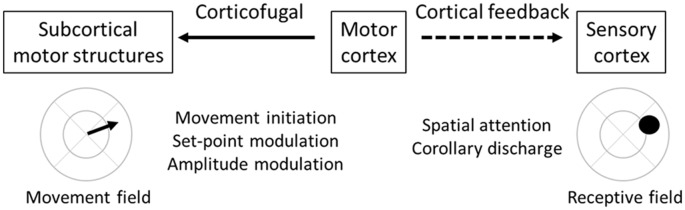
Feedforward and feedback functions of motor cortex. Motor cortex (center) projects both to subcortical structures along corticofugal pathways (solid arrow) and to sensory cortices along cortical feedback pathways (dashed arrow). These two pathways originate from different populations of motor cortex pyramidal neurons. However, within local regions of the motor cortex, there is alignment between the target movement field of the corticofugal neurons and the target receptive field of the cortical feedback neurons. Functions of the corticofugal pathway relate to motor control, including movement initiation, set-point modulation, and amplitude modulation. Functions of the cortical feedback pathway relate to spatial attention and corollary discharge.

Which pathways emanating from the motor cortex modulate movement vs. attention (Figure 3)? Corticofugal neurons projecting to subcortical structures (including the brainstem and spinal cord) most directly modulate overt movement. Spatial attention, in contrast, is believed to be mediated by cortical feedback neurons projecting from the motor to sensory cortices. For this to work, there must be alignment between motor cortex movement fields and sensory cortex receptive fields. Specifically, corticofugal neurons that direct movement into a specific location should be co-mingled with cortical feedback neurons that project to sensory cortex neurons with matching receptive fields (Figure 3). The premotor theory received a strong causal test in the non-human primate oculomotor system by Moore and colleagues. First, they demonstrated that sub-threshold (for eye movement) stimulation within the frontal eye fields (FEF) shifts attention to the movement field of that region (Moore and Fallah, 2001). Second, they demonstrated that sub-threshold stimulation enhances visual responses in visual cortex V4 when the two response fields are aligned, and suppresses visual responses when not aligned (Moore and Armstrong, 2003). These findings are consistent with spatial attention mediated by motor-to-sensory cortical feedback enhancing sensory processing in the aligned receptive field. Importantly, a recent study demonstrated spatial attention signals specifically in FEF→V4 projecting cortical feedback neurons (Merrikhi et al., 2017), further implicating cortical feedback in attentional processes.

**Figure 4:**
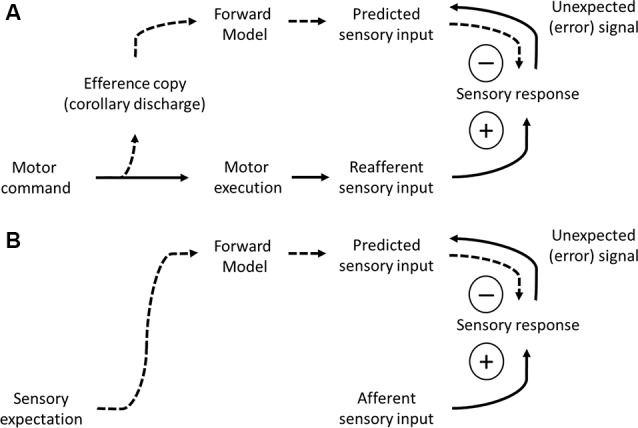
Models of predictive coding. (A) A framework of predictive coding based on movement initiation. A motor command signal (bottom left) triggers motor execution (solid arrow) and sends a copy of the motor command to sensory cortices (efference copy or corollary discharge, dashed arrow). The forward model converts the motor command to a pattern of predicted sensory input. This predicted sensory input is subtracted from the feedforward sensory input, canceling out the reafferent (self-generated) component. Unexpected signals, not predicted by self-generated movement, are not subtracted and therefore propagate forward. Efference and prediction signals are carried by feedback pathways (dashes arrows) whereas sensory signals are carried by feedforward pathways (solid arrows). **(B)** A framework of predictive coding based on sensory expectation. Sensory expectation evokes signals reflecting the pattern of predicted sensory input, to subtract from afferent sensory input. Unexpected signals, not predicted by sensory expectation, are not subtracted and therefore propagate forward. Prediction signals are carried by feedback pathways (dashes arrows) whereas sensory signals are carried by feedforward pathways (solid arrows).

The second process related to motor-to-sensory cortical feedback is corollary discharge (Crapse and Sommer, 2008). Movement evokes specific patterns of sensory inputs, due to the physical consequences of the movement itself. This is particularly problematic during active sensing, for which self-generated movement may be confused with externally generated sources. The self-generated sensory input is referred to as “reafferent,” to distinguish it from sensory signals driven by external sources (afferent). How may the brain distinguish reafferent from afferent signals? According to the theory of corollary discharge, a motor-to-sensory pathway sends a motor copy (efference) signal that informs the sensory region of an ongoing motor action. To be effective, the efference signal should cancel out the expected pattern of reafference (Figure 4A).

Studies in visual, somatosensory, and auditory cortices suggest important roles for motor-to-sensory cortical feedback in mediating an efference signal. During natural vision, saccades are frequently executed to project salient visual fields onto the fovea. If visual processing were continuous, visual percepts would blur during each saccade. However, a process known as “saccadic suppression” reduces visual processing during re-positioning of the visual field. Notably, saccadic suppression begins about 75 ms *before* eye movement, ruling out a purely bottom-up (stimulus-driven) process (Diamond et al., 2000). Reductions in visual processing associated with saccadic suppression have been observed in higher-order visual cortices and the FEF (Bremmer et al., 2009; Krock and Moore, 2016), with changes in FEF preceding reported changes visual cortices (Krock and Moore, 2016). In addition to suppression, some higher-order visual area neurons shift their receptive field focus towards the endpoint of the impending saccade before saccade generation (Tolias et al., 2001). These receptive field shifts may function to enhance visual processing within the saccade target even before fixation. Both saccadic suppression and receptive field shifts are likely driven by corollary discharge signals originating in the superior colliculus and propagated to visual cortical areas through FEF cortical feedback pathways (Sommer and Wurtz, 2004; Hamker and Zirnsak, 2006; Sommer and Wurtz, 2006; Zirnsak et al., 2014).

Active sensing is also a critical feature of the somatosensory system. We actively move our appendages to sample the local environment. This is exemplified in the rodent whisker system; rodents rhythmically whisk at a frequency of 8–10 Hz, enabling them to sample a wide range of space surrounding their head and neck (Carvell and Simons, 1995; Harvey et al., 2001). This self-generated movement activates the stretch receptors at the base of each whisker follicle, stimulating the same neural pathways that encode whisker deflections caused by extrinsic sources (Moore et al., 2015; Severson et al., 2019). How does the brain distinguish self-generated whisking from contact with external stimuli? To study this, Fee et al. (1997) blocked the facial nerve unilaterally, thereby abolishing whisking and any reafferent signals from that side of the face. During nerve block, they recorded in sensory cortex a persistent neural signal matching the whisking amplitude envelope (indexed by the unaffected, ipsilateral whisker field), consistent with an efference signal (Fee et al., 1997). The source of this presumed efference signal is likely a combination of ascending neuromodulatory input (Eggermann et al., 2014) and cortical feedback from the whisker representation of motor cortex, which encodes the initiation and amplitude modulation of whisking (Carvell et al., 1996; Hill et al., 2011; Zagha et al., 2013).

In addition to active sensing, locomotion can evoke self-generated stimuli that must be distinguished from externally generated stimuli. The two examples we describe below relate to consequences of locomotion for the auditory and visual modalities. With each footfall, walking or running creates expected patterns of auditory stimuli (i.e., walking on gravel). Top-down processes may be used to suppress these expected reafferent inputs. Recent studies in mouse have identified a motor-to-auditory cortical feedback circuit that mediates reafferent suppression (Nelson et al., 2013; Schneider et al., 2014). These studies demonstrated locomotion-related suppression of pyramidal neurons in the auditory cortex, mediated at least in part by motor cortical feedback activation of PV-containing GABAergic interneurons. Remarkably, this form of reafferent suppression is not fixed but adapts to new patterns of reafference (Schneider et al., 2018).

Regarding vision, walking or running alters the visual flows of objects in the environment in expected ways (e.g., faster locomotion causes faster visual flow). Visual and locomotion signals are integrated with mouse primary visual cortex (V1; i.e., Niell and Stryker, 2010; Keller et al., 2012; Saleem et al., 2013; Pakan et al., 2016). What is the purpose of this integration? One possibility is to enhance visual processing during locomotion to aid in spatial navigation (Niell and Stryker, 2010; Fu et al., 2014). Recent studies, however, argue for a predictive function of accounting for expected visual flows. Keller et al. (2012) demonstrated robust prediction error signals in V1, evoked by disrupting visual flow during locomotion (Keller et al., 2012). Moreover, a recent study identified cortical feedback axons from the secondary motor cortex to V1 as a source of locomotion signals (Leinweber et al., 2017). Remarkably, the authors demonstrated that motor-to-visual feedback activity adapts to learned expectations of visual flow. Thus, we find that across modalities, sensory cortices receive motor cortex feedback signals to distinguish between expected and unexpected sensory consequences of movement.

The feedback-mediated processes described in this section will amplify or suppress sensory representations according to attentional focus (for spatial attention) or behavioral context (for corollary discharge). Sensory representations within the attended field and stimuli not predicted from ongoing behavior will propagate forward to decision and motor areas, potentially triggering changes in behavior, memory, or future trigger conditions. Sensory representations in unattended fields or expected from ongoing behavior will be suppressed and less likely to exceed the trigger conditions.

### Higher Sensory Cortices to Lower Sensory Cortices (Predictive Coding and Sensory Perception)

Our final category of cortical feedback is from higher-order sensory to lower-order sensory cortices. The first process we consider is predictive coding (Figure 4B). Predictive coding is related to the discussion of corollary discharge above, in that corollary discharge is a specific form of predictive coding pertaining to self-generated movement. Here, we discuss aspects of predictive coding that are not movement-related, and therefore do not originate from the motor cortex. Predictions about sensory inputs are also derived from prior knowledge about the sensory content of our surroundings. These predictions can be at the level of stimulus features (e.g., linear stimuli in natural scenes tend to continue for some distance) or objects (e.g., when I enter my office, I should see my computer). Mumford (1992) put forward the following hypothesis regarding sensory predictions. He hypothesized that sensory predictions originate in higher-order sensory cortices and propagate along feedback pathways to lower-order sensory cortices, where they cancel out predicted patterns of sensory inputs. Following this computation (subtraction of predicted signals from the sensory input) what remains is a prediction error signal, reflecting stimulus features that were not expected (Figure 4B). The error signal propagates along feedforward pathways back to the higher-order sensory cortices. If the error is strong enough, it can provoke a new prediction (e.g., I opened the door to the wrong office!). This process of predictive coding is iterative and dynamic until it converges on a solution in which expectation matches sensory evidence (Mumford, 1992).

The first component of Mumford’s hypothesis, that cortical feedback suppresses expected stimulus responses, has received support from computational simulations and physiological studies. Rao and Ballard (1999) created a hierarchical neural network with feedforward and feedback connections and increasing receptive field size (as in hierarchical cortical networks). The network was trained on natural images to optimize the ability to predict the stimulus drive at each level (minimize the difference between feedforward stimulus responses and feedback predictions). Interestingly, these simulations produced model neurons with many classical and extra-classical receptive field-like properties, such as end-stopping (Rao and Ballard, 1999). The similarities of these models with the cortical visual system argue for predictive coding as an organizing principle of cortical circuits, with a specific role for cortical feedback in canceling expected stimulus responses from lower-order regions. Some of these findings have now been tested experimentally in the primate visual system. Notably, suppressing higher-order cortical areas reduced surround suppression and end-stopping in the primary visual cortex (Hupe et al., 1998; Nassi et al., 2013), as hypothesized by the predictive coding framework. Importantly, specific involvement of cortical feedback has recently been confirmed, by selectively inhibiting the V2-V1 feedback pathway and demonstrating reduced surround suppression in V1 (Nurminen et al., 2018).

The second component of Mumford’s hypothesis predicts a dynamic interplay between feedback prediction and feedforward sensory evidence to accomplish object or scene identification. Experimental and computational studies provide strong, yet indirect, support for this proposal. Sensory responses in primary sensory cortices differ depending on whether a stimulus is perceived (Super et al., 2001; see also Cauller and Kulics, 1988). Neural correlates of perception do not impact the initial sensory responses but emerge tens of milliseconds later, consistent with a feedback source (Pascual-Leone and Walsh, 2001). Why does sensory perception require cortical feedback? We consider four possible theories. One theory argues that while feedforward propagation is sufficient for generalized, categorical perception, feedback is required to resolve detailed stimulus features (Lamme and Roelfsema, 2000; Hochstein and Ahissar, 2002). According to this theory, feedback is required to focus attention on salient receptive fields for fine discrimination. A hierarchical neural circuit model demonstrated feasibility for this theory and was able to distinguish between detailed stimuli only after several cycles of feedforward and feedback signaling (Jehee et al., 2007). A second theory argues that feedforward models alone are insufficient for object recognition due to the inability to uniquely determine the cause of sensory input without making a set of assumptions or inferences (e.g., that dress was white and gold, not blue and brown). According to this theory, cortical feedback provides the context-dependent inferences required for unique identification (Friston, 2003). A third theory proposes that cortical feedback contributes to perception by altering the receptive field properties of lower-order neurons, as needed for a given task (Gilbert and Li, 2013). A fourth theory proposes that identification emerges from coincident input onto pyramidal neuron dendrites: feedforward sensory evidence targeting proximal dendrites and feedback predictions targeting apical dendrites (Xu et al., 2012; Larkum, 2013; further described below). It is not clear yet how these perceptual processes relate to signal-routing. However, it brings up the intriguing possibility that just like motor initiation, perceptual awareness may also be regulated by trigger conditions which require the interplay of feedforward and feedback signaling.

### Overview and Generalization

As we have seen above, cortical feedback pathways distribute contextual signals throughout the cortex. Specific pathways may contribute to motor planning, impulse control, spatial attention, corollary discharge, predictive coding, or sensory perception. However, instead of considering these as isolated processes, we propose that cortical feedback pathways work together to regulate feedforward signaling to promote goal-directed behavior. We consider the “starting-point” for this process to be the PFC, for its ability to maintain a representation of one’s current goals through working memory. We propose that through cortical feedback, the PFC sets the trigger conditions for the outputs of cortex: motor initiation, memory formation, and decision-making. Moreover, as we’ve seen for motor cortices, feedback from the PFC can prepare specific outcomes (e.g., left arm reach or running from a starting line) to be deployed once the trigger conditions are met. Concurrently, feedback to sensory cortices can amplify or suppress sensory representations according to goal-direction and behavioral or environmental context. Processes of attention and predictive coding enhance attended and unexpected sensory representations and suppress unattended and expected sensory representations. Stronger sensory representations have a higher probability of exceeding trigger conditions and thereby altering behavior and cognition; weaker sensory representations have a lower probability of exceeding trigger conditions and thereby are more likely to be ignored. Changes in goal-direction mean new goal representation in the PFC, which again propagates along feedback pathways to favor new sensory and motor trajectories. By regulating the initiation and propagation of stimulus and response signals, cortical feedback can form dynamic, arbitrary, context-dependent sensory-motor associations.

## Cellular Mechanisms of Cortical Feedback

**Figure 5:**
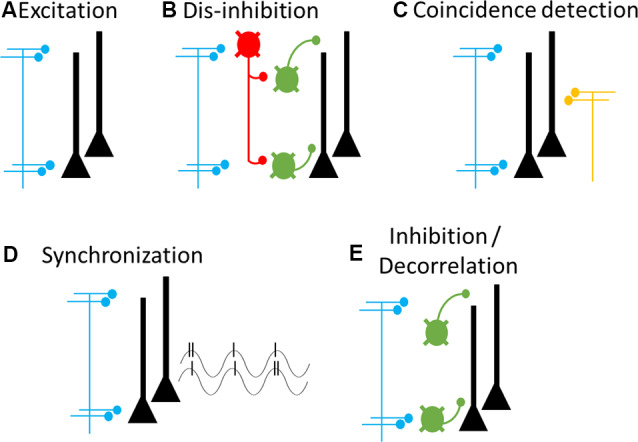
Cellular and circuit mechanisms of cortical feedback modulation. Proposed cellular targets and mechanisms of cortical feedback pathways. Blue axons and terminals represent cortical feedback pathways, yellow axons and terminals represent feedforward pathways. Neurons in the target area include pyramidal neurons (black) and multiple types of GABAergic interneurons (green and red). **(A)** Cortical feedback forms excitatory synapses onto pyramidal neurons, leading to increased excitation. **(B)** Cortical feedback synapses onto interneuron-targeting GABAergic interneurons (red), leading to dis-inhibition. **(C)** Coincident inputs from feedforward and feedback pathways lead to dendritic spiking and supra-linear increases in pyramidal neuron output. **(D)** Phasic activity of cortical feedback leads to pyramidal neuron synchronization, both locally (shown here) and between source and target regions (not shown). Synchronization can ensure optimal post-synaptic integration and thereby enhance coupling among synchronized regions. **(E)** Cortical feedback synapses onto pyramidal neuron-targeting GABAergic interneurons, leading to increased inhibition and pyramidal neuron decorrelation. It is likely that any feedback pathway may deploy any combination of these mechanisms, as needed for a specific goal or context.

We next, consider the cellular and synaptic properties that underlie cortical feedback function. Cortical feedback pathways form excitatory synapses onto many neuronal populations within the cortical circuit (Figure 5). This includes pyramidal neurons and multiple subtypes of GABAergic interneurons such as those containing parvalbumin (PV+), somatostatin (SOM+), and vasoactive intestinal peptide (VIP+; Gonchar and Burkhalter, 2003; Lee et al., 2013; Zagha et al., 2013; Kinnischtzke et al., 2014). None of these populations appear to be exclusively targeted by feedback, compared to feedforward and thalamic inputs, although patterns of innervation will differ depending on the laminar distribution (Tremblay et al., 2016). Whereas PV+ and SOM+ interneurons strongly inhibit pyramidal neurons, VIP+ interneurons preferentially target other GABAergic interneurons which leads to pyramidal neuron disinhibition. Thus, cortical feedback may exert its effects within its target region by increasing excitation, increasing inhibition, or through VIP-mediated disinhibition. Moreover, by modulating excitation and inhibition, cortical feedback may also alter the coherent population dynamics (i.e., “state”) of its target region (Zagha et al., 2013; Zagha and McCormick, 2014; Zagha et al., 2016).

How do these cellular targets underlie the cognitive and behavioral functions described above? We consider five different mechanisms. The first four cellular mechanisms relate to enhancing activity in the target region, as required for attention and motor preparation, whereas the fifth mechanism relates to suppressing target activity, as required for predictive coding and motor inhibition. First, cortical feedback may directly depolarize pyramidal neurons (Rocco and Brumberg, 2007; Lee et al., 2008; Petreanu et al., 2009; Zagha et al., 2013; Kinnischtzke et al., 2014), thereby increasing spontaneous and sensory-evoked spike rates of pyramidal neurons within the targeted receptive or movement field (Figure 5A). Second, cortical feedback may indirectly depolarize pyramidal neurons by stimulating VIP-containing GABAergic interneurons, which would dis-inhibit pyramidal neurons (Lee et al., 2013; Zhang et al., 2014; Figure 5B). A third possible mechanism involves the intrinsic excitability of pyramidal neuron dendrites. Inputs into the apical dendrites of pyramidal neurons alone have little impact on action potential spiking, due to the large electrotonic distance to the site of spike generation in the axon initial segment. However, Larkum et al. (1999) found that co-incident inputs to the soma and apical dendrites can drive dendritic Ca^2+^ spikes and supra-linear increases in action potential output. Such co-incident input could come from cortical feedback (targeting apical dendrites) and feedforward inputs (targeting proximal dendrites; Cauller, 1995; Xu et al., 2012; Figure 5C). This organization would predict cortical feedback alone to have little impact on spontaneous spiking but substantially increase sensory-evoked responses. Consistent with this prediction, in the oculomotor visual system, the effects of FEF stimulation on sensory cortex neurons are minimal without co-incident sensory stimuli (Moore and Armstrong, 2003). Depolarization, through either of these mechanisms, may be highly effective in enhancing cortical coupling. Due to non-linear membrane dynamics, modest depolarizations can result in multiplicative increases in a neuron’s input-output function (Murphy and Miller, 2003). Connected networks with high input-output gain will display enhanced signal propagation compared to connected networks with low input-output gain (Haider and McCormick, 2009). Fourth, cortical feedback may synchronize the neurons within attended or preferred signaling pathways (Fries et al., 2001; Figure 5D). Synchronization can enhance post-synaptic integration and thereby increase coupling strength between cortical regions, without necessarily increasing spontaneous or sensory-evoked spike rates from lower-order neurons. The fifth mechanism relates to suppressing activity in the target region. Suppression may be mediated by the activation of PV+ or SOM+ interneurons, which directly inhibit pyramidal neurons (Figure 5E). Importantly, inhibition may also improve the specificity of signal processing by decorrelating nearby pyramidal neurons (Renart et al., 2010; Zagha et al., 2013, 2016). Overall, we recognize that cortical feedback has a diverse yet limited repertoire of cellular and circuit mechanisms by which to exert its behavioral and cognitive functions.

## Shaping the Cortical Landscape

Putting it all together, we propose that cortical feedback carries out its cognitive and behavioral functions by routing sensory and motor signals according to goal-direction and context. At the cellular level, this is implemented by modulating patterns of excitation and inhibition in the target regions, thereby enhancing certain neural ensembles and suppressing others. By establishing these patterns across multiple hierarchical levels, cortical feedback determines the content, threshold, and direction of feedforward propagation. In this last section, we consider theoretical and experimental approaches that may be applied to studying dynamic signal routing in the cortex.

**Figure 6:**
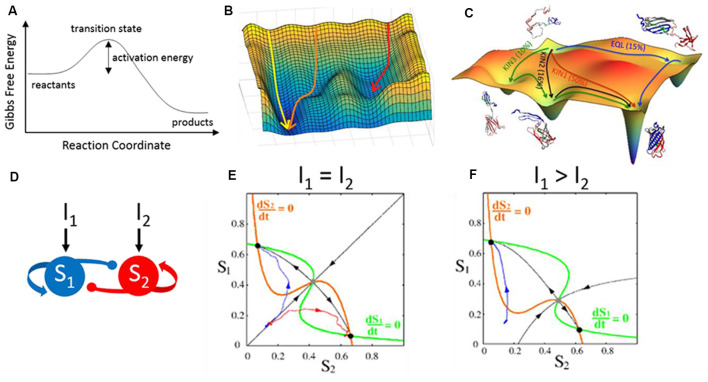
Energy landscapes across disciplines. (A) Gibbs free energy curve illustrating the transformation of reactants to products across an energetically unfavorable transition state. This curve depicts a reaction occurring within a single dimension. **(B)** Reactions occurring within two dimensions can be depicted as an energy landscape or potential energy surface. Shown here are three simulations (arrows) along a hypothetical two-dimensional energy landscape. The initial conditions and contours of the landscape determine the simulation trajectories, which flow from states of high (peaks) to low (troughs) free energy. **(C)** A simulated energy landscape for the re-folding of denatured green fluorescent protein (GFP), with multiple potential pathways (arrows) undertaking different partial folding states. **(D–F)** An analogous form of energy landscapes, used here to model neural circuit activity. **(D)** The structure of the circuit model is two populations (S1 and S2) which self-excite (arrows) and cross-inhibit (circles). Each population can be driven by external inputs (I1 and I2). **(E)** A phase plane representation of the activities of S1 vs. S2 provides an activity landscape of the network. Critical features of this landscape are the fixed points demarcated at the crossings of the nullclines (orange and green lines). Stable fixed points (black dots) are analogous to troughs in an energy landscape. The unstable fixed point in this example (gray dot) would have the shape of a saddle in an energy landscape: sloping down *towards* the fixed point (black arrows towards) and sloping down *away* from the fixed point (black arrows away). The two simulations shown in blue and red have the same starting point but diverge from each other and converge into the two stable fixed points. **(F)** Changing the inputs alters the landscape, which now (I1 > I2) favors activity trajectories towards the top-left fixed point. Panel **(C)** is reproduced with permission from Reddy et al. (2012), panels **(E,F)** are reproduced with permission from Wong and Wang (2006), copyright 2006 Society for Neuroscience.

A potentially useful framework to consider is transition state theory as developed in physics and chemistry. Chemical reactions transform chemicals from reactants to products across energetically unfavorable intermediate transition states. The height of the transition state determines the activation energy needed to complete the reaction. For chemical reactions within a single dimension, this can be demonstrated in a free energy curve (Figure 6A). We can develop an analogy to signal routing in the cortex if we replace “reactants” with “sensory signals” and “products” with “motor outcomes.” The height of the transition state would reflect the trigger conditions required for a sensory signal to initiate a motor response. For any specific sensory-motor pairing, cortical feedback can modulate the height of the activation energy barrier to prevent (higher) or facilitate (lower) successful sensory-motor triggering.

However, natural behaviors, like natural chemical reactions, do not exist in a single dimension. To capture higher-order dimensions, we can depict a two-dimensional energy landscape (also called a potential energy surface; Figures 6B,C). Like the free energy curve, peaks in the landscape are energetically unfavorable. All troughs in the landscape are locally favorable, whereas the deepest trough is the most favorable solution. What makes a landscape useful is that one can visualize the current state of a system in the context of all possible states and the likelihood of transitioning between states. For example, a protein may fold in many different configurations. An energy landscape can demonstrate which configurations are stable (valleys in a Gibbs free energy landscape) and the amount of energy required to transition between configurations (peaks in the landscape; Figure 6C). Different types of landscapes are currently used in neuroscience research. Phase plane analyses are used to visualize the excitability of model neurons (Rinzel and Ermentrout, 1998) and the activity trajectories of simulated neural circuits (Wong and Wang, 2006; Figures 6D–F). Fixed points and flow lines illustrate how the system will evolve as new stimuli arrive. Similarly, the Hopfield network used to model memory sequences in the hippocampus consists of stable, low energy configurations and higher energy transition states in between (Hopfield, 1982; Lisman, 1999). In each case, the landscape provides a concise yet comprehensive view of the types of activity patterns afforded by the organization of the system.

**Figure 7:**
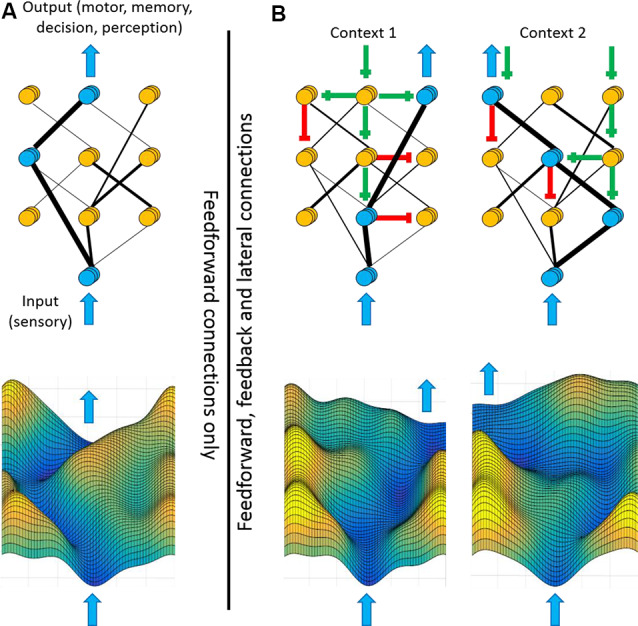
Feedback shapes the cortical landscape according to context. Framework for how feedback pathways may contribute to flexible sensory-motor processing. Top panels illustrate hierarchically-arranged cortical nodes with input regions at the bottom and output regions at the top. Blue arrows represent sensory inputs (bottom) or cortical outputs (top). Blue nodes represent highly active regions whereas yellow nodes represent less active regions. Black lines represent the structural and functional connectivity between nodes, with thicker lines depicting stronger connectivity. Bottom panels are energy landscape representations of the above networks. The height of each contour reflects the energy barrier of propagating into that region. **(A)** If signal processing between cortical regions is defined only by feedforward connections, then sensory-motor processing will always follow the strongest connections. **(B)** Different contexts, such as goal-direction or self-generated movement, can propagate along feedback and laterals pathways [green lines (excitatory) and red lines (inhibitory)]. Consequently, these top-down signals modulate which input and output nodes are enhanced or suppressed, thereby altering the coupling strengths between cortical regions (black lines). The same input signal may now be routed to diverse outputs, as appropriate for the current context. As illustrated in the bottom panels, modulations of activity levels within individual regions and coupling strengths between regions change the contours of the cortical landscape, favoring different input-output trajectories.

We propose considering the cortical sheet as a two-dimensional energy landscape (Figure 7). Consider sensory inputs arriving at the bottom of the landscape and outputs (motor, memory, decision, and perception) emerging from the top. The value of the landscape at each point reflects the cost (or energy barrier) of feedforward propagation into that area. The contours of the landscape reflect both the structural constraints of cortical anatomy and cortical feedback modulations of sensory/motor representations and coupling strengths. Peaks separating sensory inputs from outputs reflect the triggering conditions, with higher peaks preventing unwanted sensory-motor associations. Valleys in the output regions reflect intended actions whereas hills in the input regions reflect expected sensory representations. Channels connecting cortices reflect enhanced coupling and preferred routes of propagation. As illustrated in Figure 7, a circuit with only feedforward pathways restricts signal propagation to the strongest anatomical connections. The inclusion of feedback pathways (Figure 7B) changes the coupling between cortical regions, thereby allowing diverse patterns of signal propagation and an array of sensory-motor associations. Feedback to sensory cortices creates channels along attended sensory streams towards the output regions. Feedback to motor cortices lowers the peaks (trigger conditions/activation energy) in the trajectories of intended motor plans. Given that contextual signals are mediated by synaptic excitation and inhibition, reconfiguration of the entire landscape may occur on the timescale of tens of milliseconds.

Is it possible to experimentally resolve the cortical landscape? In the section above on feature-based attention, we discussed attempts to quantify changes in coupling strengths between cortical regions with changes in attention. Such efforts are beginning to reveal the dynamic nature of the cortical landscape. So far, the dominant technologies used for these efforts are fMRI and population-level electrophysiology. Reported changes in coupling tend to be small (when quantified by a correlation coefficient, changes are typically lower than 0.2). This may reflect a dominant contribution of stable neuroanatomy to coupling strength, or rather the limitations of these recording techniques. Novel approaches, incorporating both correlational and causal methods, are needed to examine the cortical landscape at much higher spatio-temporal resolution. Additionally, once the data are collected, we will need novel analytical approaches to quantify and interpret changes to the landscape.

## Discussion

Neocortex enables us to have selective and meaningful interactions with our surroundings. Central to this ability is the contribution of cortical feedback pathways. In this manuscript, we detail ongoing efforts to study cortical feedback at the behavioral, systems, and cellular levels of organization: contributions to specific cognitive and behavioral processes, impacts on sensory and motor signaling, and cellular targets and mechanisms. To link these levels of organization we propose the framework of the cortical landscape, a depiction of the routes through cortex that connect sensory inputs to motor, memory, decision, and perception outputs. Within this framework, it is the function of cortical feedback to proactively shape the cortical landscape, to establish routes between cortical inputs and outputs as needed for one’s current goal-direction and behavioral and environmental context. Within this expanded framework of signal routing, we recognize the following directions for future research.

(1) Resolving the cortical landscape. New experimental and analytical approaches are needed to directly measure cortical physiology from the perspective of routing. This requires tools with high spatio-temporal resolution and a neocortex-wide field of view, applied to awake subjects performing goal-directed tasks. Additionally, we emphasize the need to study functional coupling throughout the cortex before stimulus presentation. What are the initial conditions within the cortex that proactively regulate the propagation of attended vs. unattended stimuli, toward intended vs. unintended action representations? Studies of energy landscapes in other disciplines may be particularly useful in applying such concepts to cortical physiology. With these tools, we will then be able to test how various patterns of excitation and inhibition regulate signal propagation to influence behavior and cognition.
(2) Specific perturbations of cortical feedback. Most studies to date have inferred functions of cortical feedback by stimulating or suppressing a higher-order cortical region thereby perturbing cortical feedback, yet also perturbing all other projection pathways. We now have the tools to selectively inhibit cortical feedback, with millisecond temporal resolution in awake, behaving subjects. Such studies are critical for determining the specific functions and mechanisms of cortical feedback pathways.
(3) Combining (1) and (2) will enable direct tests of our central theory, that cortical feedback pathways contribute to goal-directed behavior by proactively shaping the cortical landscape according to task demands.
(4) Cellular representations of context. In this manuscript, we discussed many different forms of contextual signaling. Each of these processes make testable predictions of their cellular implementation. For example, for predictive coding processes, the feedback signals should preferentially stimulate inhibitory interneurons and in turn suppress activity in neurons tuned to expected sensory representations (i.e., Schneider et al., 2018). How do different circuit motifs contribute to different contextual signaling processes? Is there a core “prediction circuit” or “attention circuit” (Batista-Brito et al., 2018)?
(5) Development and refinement of cortical feedback pathways. Cortical feedforward and feedback pathways show different patterns of postnatal development (Barone et al., 1995; Batardière et al., 2002; Dong et al., 2004). While much research to date has explored the mechanisms of development and plasticity of feedforward pathways, we know very little about similar processes for feedback pathways. Does cortical feedback development and plasticity account for the acquisition and learning of specific cognitive processes?
(6) Contributions of cortical feedback to neuropsychiatric disease. The research described above suggests that impaired cortical feedback would manifest as impulsivity, inattention, and impaired expectations/perceptions. Such cognitive, behavioral, and perceptual disturbances are prominent features of many neuropsychiatric conditions, including ADHD, autism, and schizophrenia (Schachar et al., 1995; Uhlhaas and Mishara, 2007; Robbins et al., 2012). However, it is entirely unknown the extent to which impaired cortical feedback contributes to the signs and symptoms of neuropsychiatric disease.

## Author Contributions

EZ conducted the research and wrote the manuscript.

## Conflict of Interest

The authors declare that the research was conducted in the absence of any commercial or financial relationships that could be construed as a potential conflict of interest.
