# Addendum: Shaping the Cortical Landscape: Functions and Mechanisms of Top-Down Cortical Feedback Pathways

**DOI:** 10.3389/fnsys.2020.632485

**Published:** 2020-12-11

**Authors:** Edward Zagha

**Affiliations:** Neuroscience Graduate Program, Department of Psychology, University of California, Riverside, Riverside, CA, United States

**Keywords:** feedback, motor preparation, attention, predictive coding, cortical circuits, functional connectivity

Due to the addition of the published article to the Research Topic “Feed-forward and Feed-back Processing in the Cerebral Cortex: Connectivity and Function,” the inclusion of the following **Transparency Statement** is required to ensure full transparency of the previous review process and to prevent any perception of a conflict of interest.

“*This manuscript was published prior to the launch of the Research Topic” Feed-forward and Feed-back Processing in the Cerebral Cortex: Connectivity and Function “for which the handling Editor and the author are co-Editors. The handling Editor and the author confirm the absence of any collaboration during its review process.”*

